# The Paramak: automated parametric geometry construction for fusion reactor designs.

**DOI:** 10.12688/f1000research.28224.1

**Published:** 2021-01-18

**Authors:** Jonathan Shimwell, John Billingsley, Rémi Delaporte-Mathurin, Declan Morbey, Matthew Bluteau, Patrick Shriwise, Andrew Davis

**Affiliations:** 1 Culham Centre for Fusion Energy (CCFE), Culham Science Centre, Abingdon, OX14 3DB, UK; 2 CEA / IRFM, F-13108 Saint-Paul-lez-Durance, F-13108 Saint-Paul-lez-Durance, France; 3 Laboratoire des Sciences des Procédés et des Matériaux, LSPM, UPR3407, CNRS, Université Sorbonne Paris Nord, Villetaneuse, F-93430, France; 4 Department of Physics, Swansea University, Swansea, SA2 8PP, UK; 5 Argonne National Laboratory, Lemont, IL, 60439, USA

**Keywords:** parametric, design, automated, fusion, energy, CAD

## Abstract

During the conceptual design process of fusion reactors it is useful to rapidly prototype different design concepts and assess their suitability against a range of high level requirements. Rapid prototyping allows the 'fail early' mantra of other fields to be applied to engineering design.

Furthermore, the rapid generation of low fidelity analysis allows fast exploration of design space, which enables better decisions to be made during concept selection and the detailed design phase. The Paramak is an open-source tool that aims to provide automated parameter driven 3D CAD models for fusion reactor components and magnetic fusion reactors. The geometry produced is compatible with several analysis workflows and this allows iterative automated model building and analysis to help steer the design concept optimisation process. The Paramak uses CadQuery 2 to create the 3D CAD model. The Paramak framework is used to create a few example reactor configurations including: a spherical reactor, a regular large radius tokamak and a compact submersion tank reactor. Input parameters for the various reactors that the Paramak can generate generally fall into three categories: continuous ranges such as blanket thickness, integer ranges such as number of toroidal field coils and categorical parameters such as type of divertor. The Paramak facilitates parameter studies where users can investigate the impact of input design parameters on the reactor performance. The use of modern software practices allows the geometry to be continuously tested in analysis workflows to ensure it is fit for purpose. The generation of output metrics from input parameters lends itself to the use of data science and machine learning approaches in order to steer the design. The Paramak provides rapid construction of analysis ready CAD in a manner that allows the designer to save time when exploring the design space for design studies and facilitate automated generative design.

## Introduction

When assessing the suitability of a fusion reactor design, one of the stages is the construction of a 3D model. This tends to be a digital 3D CAD model which is then adapted for use in different analysis disciplines, for example, engineering and neutronics. Following the conclusion of analysis, feedback can be provided and the design cycle can be iterated to refine and optimise the design. Automating the analysis can help to rapidly develop a design as shown in 1. While some automated analysis remains a challenge in certain disciplines, progress is being made on the automation of a number of domains used in the analysis of fusion reactors. In domains such as aerospace ^2^, the automatic generation of simplified representative CAD geometries has given great benefit to the concept design process. Fusion reactor design processes involve analysis being carried out and fed back into the creation of the next CAD model; this process is usually a manual GUI based operation. The interpretation of results from analysis and geometric modifications are necessarily decoupled in this mode of operation, which slows the speed of iteration. The situation can occur where the models are updated several times as different analysis streams feedback into the design. In the case where different analysis tasks take different amounts of time, there may be situations where the analysis streams therefore get different versions of geometry, before it is further modified for their respective analysis. The scripts that users create to generate the CAD model are compatible with version control and therefore the method of creating the CAD can be version controlled and traced across a design process. Having this automated model creation for simple space reserving could be considered the first stage in creating a more efficient, automated, rapid and reproducible design cycle. Automated model creation can reduce the risk of geometry creation becoming a bottleneck in the design cycle. While complex model construction might be difficult to automate with the current software, there is utility in automating simpler models and allowing the analysis of specific geometry details to be filled in at a later stage. Additionally, there is also some utility in the use of automated CAD in conjunction with automated analysis at early stages in the design, where simple models are more appropriate.

A key advantage of creating a 3D reactor geometry from parameters is that the produced model then becomes easy to quantify in terms of a small set of values. Being able to describe a 3D model with a series of parameters allows direct linking between an optimiser, input parameters and output metrics. The designer’s input is still required to make the parameterisation rules that allow components to be varied in ways that impact their performance. A designer’s skill is required to ensure the layout of components interface correctly and do not overlap. A benefit of the parametric model construction process is that when one parametric model is made this results in many perturbations that can be generated by scripts, while a static model remains a single static model. A disadvantage is that creating a parameterised model layout is more complex than a single model.

## Approach

The efficient use of CAD in the design process requires a well thought out and coherent approach to utilise all the potential benefits. While there are many possible approaches to the challenge, the Paramak focuses on an automated, parameter driven, permissive and open-source approach. This section aims to justify that decision.

### CadQuery

CadQuery 2 ^3^ offers a potential solution for the creation of automated parametric CAD. CadQuery 2 is an open-source Python library that binds to OpenCascade (OCCT 7.5 release) ^4^ and has some unique features among the possible open-source candidates. One such capability is the ability to search, filter and then operate on the CAD solids during construction. This allows components to be linked and built from each other without the user having to be concerned with redefining related solids when a linked solid is modified. This is already possible with proprietary CAD software but these capabilities are now emerging into the open-source area. CadQuery development is ongoing and the specific version used for this publication is 5.

### Integration in workflows

When using a complete CAD and analysis solution that includes a parametric CAD modelling package, the transfer of parametric models from the CAD engine to the analysis software can be achieved. Within the ecosystem of commercial PLM (Product Lifecycle Management) systems it is entirely possible to generate parametric CAD and use it within parametric analysis workflows. When wishing to utilise the CAD geometry in external analysis workflows that are not included within the ecosystem of proprietary software solutions, the use of parametric CAD becomes more challenging. The provision of CAD models via open formats such as STP format AP214 ^6^ do not support the encoding of parametric components within the CAD file. This lack of parametric support in the file formats that are used for transferring the model results in non parametric CAD being received at the analysis level for certain types of analysis. There are several possible solutions for this such as incorporating more analysis into the PLM software or developing new open CAD formats that support parametric components such as STP AP242 ^7^. The approach taken by the Paramak is to provide a parametric creation of non parametric STP files with a permissive licensing model. The combination of permissive licensing and parametric studies allows automated geometry creation and analysis to be carried out on potentially tens of thousands of designs in parallel. Cloud bursting together with Cloud computing can provide the computing resources for such a study. Traditional licensing models where the costs scale with number of parallel sessions can result in significant costs implications for such a spike in compute capacity. Depending on the number of parallel sessions required these licensing costs can become significant. Permissively licensed open-source software offers a solution to such a scenario. Since the Paramak is distributed with a permissive usage MIT open-source licence, it is therefore compatible with cases where parallel sessions are desired without incurring any licensing costs. As cloud computing grows both in popularity and market penetration, the licensing of software becomes an important factor in the software’s utility. This is reflected by the growing popularity of permissive open-source licensing ^8^.

### Software practices employed

The source-code is under version control and openly available via Github ^9^ under a permissive MIT licence. The Paramak Python package is distributed via PyPi ^10^ and there are plans to incorporate a Conda distribution in the future. A containerised build environment is distributed via Dockerhub ^11^ containing a pre-built environment with all the required dependencies. The code is documented with diagrams and examples on ReadTheDocs ^12^ which makes use of extensive Docstrings within the code. The code has been internally reviewed by a Research Software Engineer internal to UKAEA and also undergoing a professionally reviewed by an external company PullRequest ^13^. Continuous integration has been implemented using CircleCI ^14^ to run a broad range of unit tests and integration tests. The test suite also covers use of the parametrically generated CAD in neutronics simulations using DAGMC ^15^ and OpenMC ^16^. This helps ensure the geometry made is suitable for use in neutronics analysis. Github Actions have been utilised from an early stage for automating several aspects of the code distribution, packaging and static code analysis. Github Actions have been used for employing code style guides (PEP8), updating the PyPi package distribution and automatically building and uploading new Dockerhub images with each new version of the code. The decision to open-source the Paramak code was a key enabler that allowed use of the previously mentioned platforms and in turn allowed the code to grow and improve rapidly. Additionally, the open-source nature of the project has facilitated contributions from outside the organisation, as demonstrated by the wide author list and contributors on to the Github repository.

## Code structure and examples

**Figure 1.**
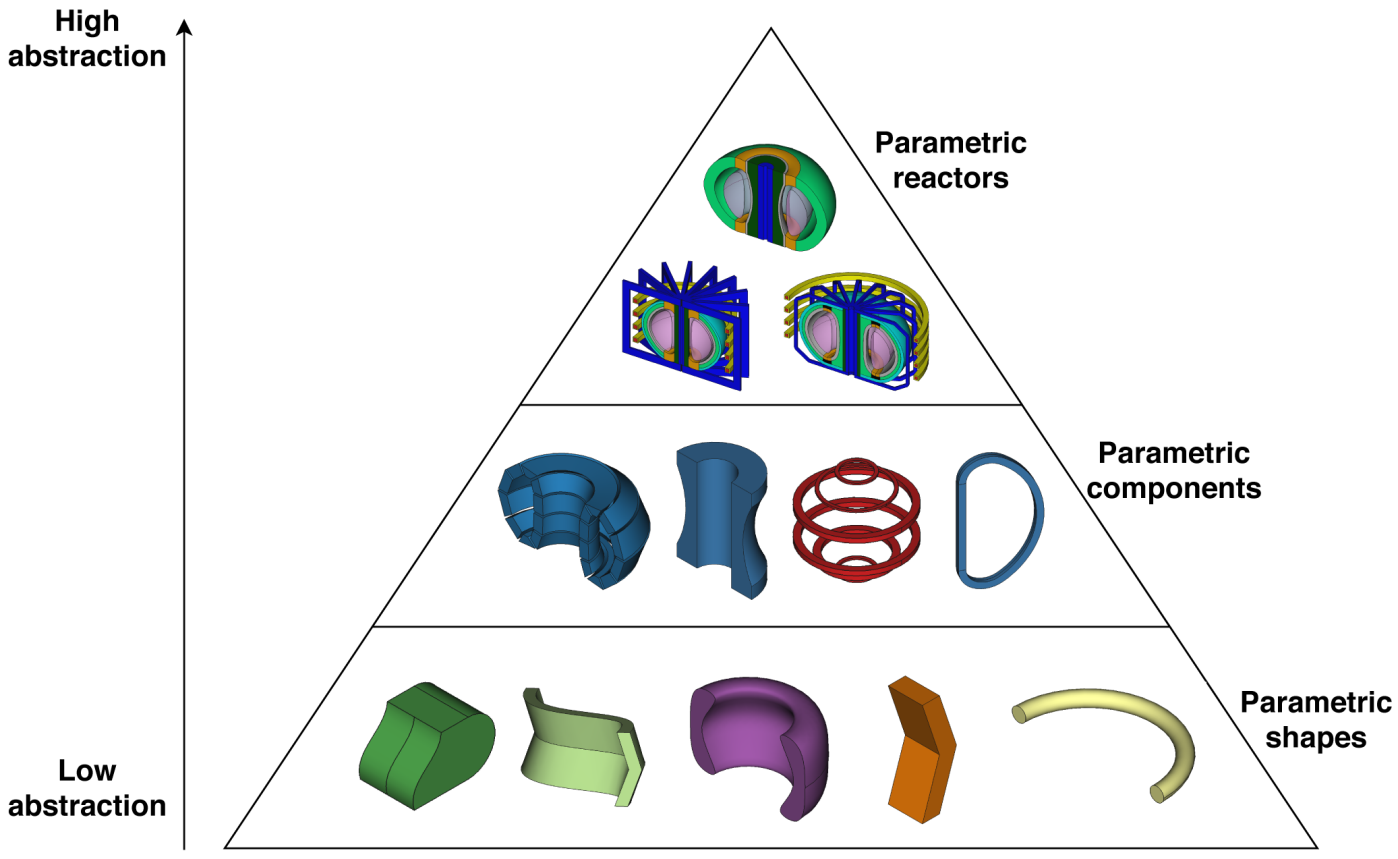
Representation of the Paramak structure showing examples of Parametric Shapes, Parametric Components and Parametric Reactors.

The Paramak consists of three main groups of classes: Shapes, Components and Reactors (see Figure 1).

### Parametric Shapes

**Figure 2.**
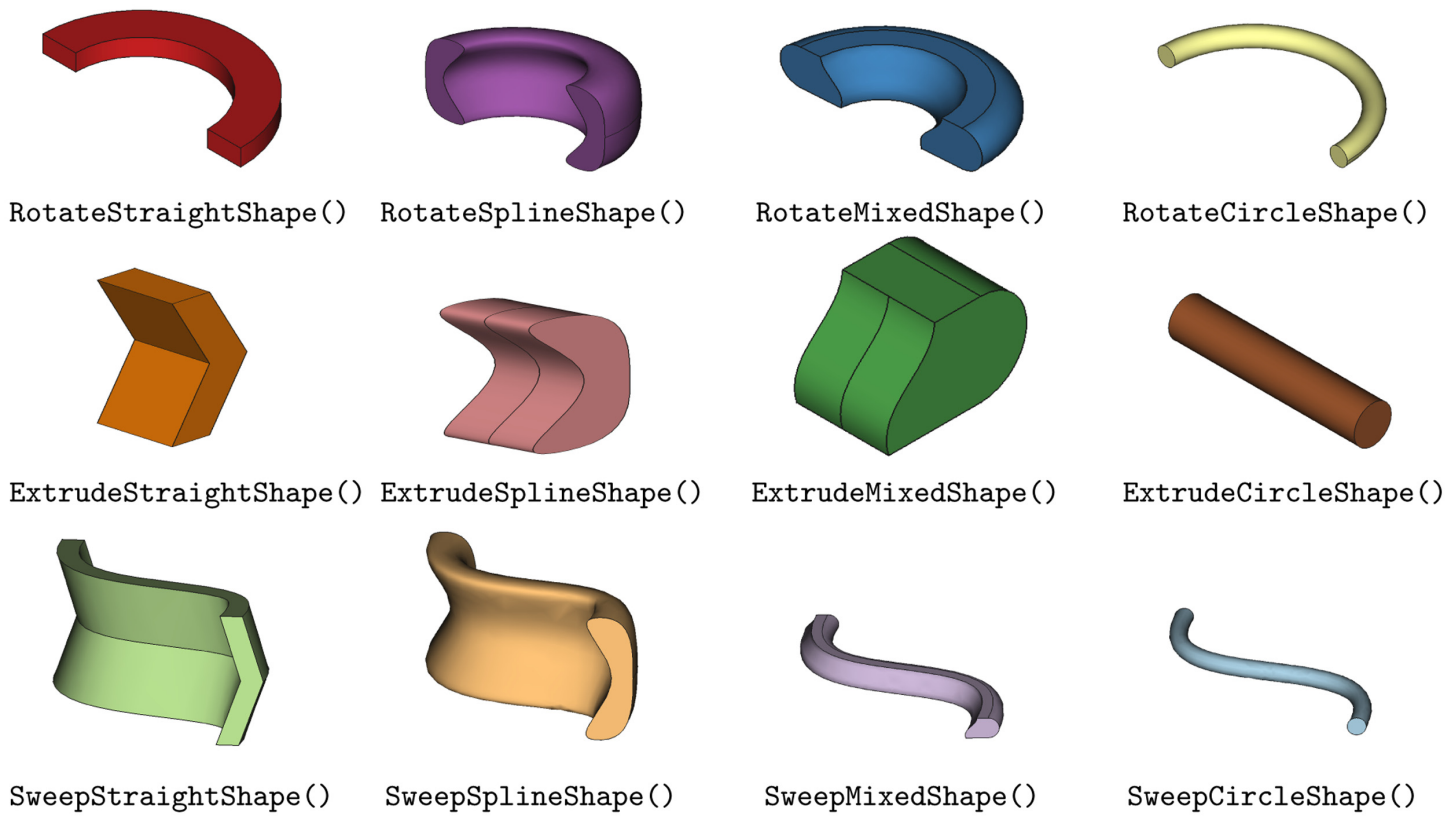
Primitive Shape Classes which require the user to specify input points.

The Parametric Shapes provide profiles from a combination of straight edges, circular edges and Bezier spline edges. These shapes can represent a wide range of basic shapes and are made from a series of 2D coordinates. Shapes can be operated on to create 3D volumes using extrude, revolve, sweep and rotate operations (see Figure 2). Boolean operations such as cut, intersect and union are also available to Shapes. To build Shapes the class must be provided with coordinates or points and edge connection types to connect each coordinate.

### Parametric Components

The Parametric Components inherit from Shape and build upon these basic families of shapes to create volumes that more closely resemble components found in fusion reactors. Parametric Components generally have particular methods of finding the coordinates that make up the shape and are thus able to provide the coordinates needed to make a Shape class. The methods of finding points differ from component to component and are encoded within the component’s class.

For the simplest Parametric Components such as CenterColumnCylinder() the points coordinates are found based on a hollow cylinder. This requires just four points and uses straight lines to connect the points followed by a rotation around the Z axis. This is then abstracted for the user so that only the height, inner radius and outer radius are required. The Component class then finds the points from the internal rules and applies any CAD operations or Boolean operations. More complicated shapes such as the BlanketFP() (see Figure 6) finds points on the front surfaces using a variable offset from the plasma. A variable thickness between the interior and exterior surface is then used to find the rear surface points. The front and rear surface points are connected with a series of splines with straight connections between the two surfaces. The variable offsets and thicknesses can be provided as a function of poloidal angle and the component is therefore able to construct more complex 3D objects. Some components (e.g. InnerFirstwallFCCS()) are constructed entirely from other components, in this case finding their coordinates is not necessary as a surface offset and Boolean cut is sufficient to find the 3D volume.

**Figure 3.**
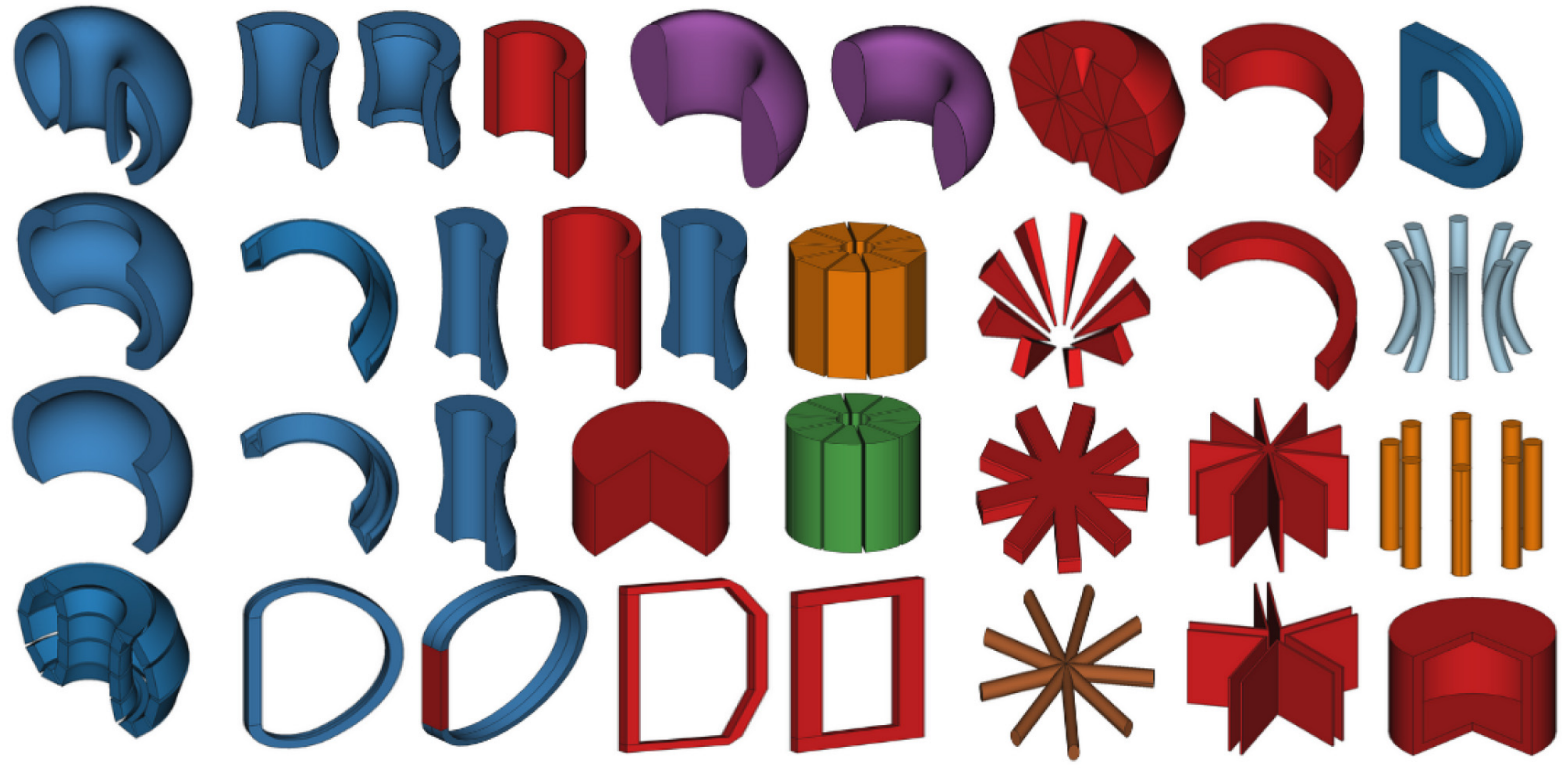
The current selection of Parametric Components available. Note that because these shapes are all customisable with parameters they can appear differently to their default view pictured in the diagram. These inherit from the Shape class and have encoded methods of calculating the points required.

**Figure 4.**
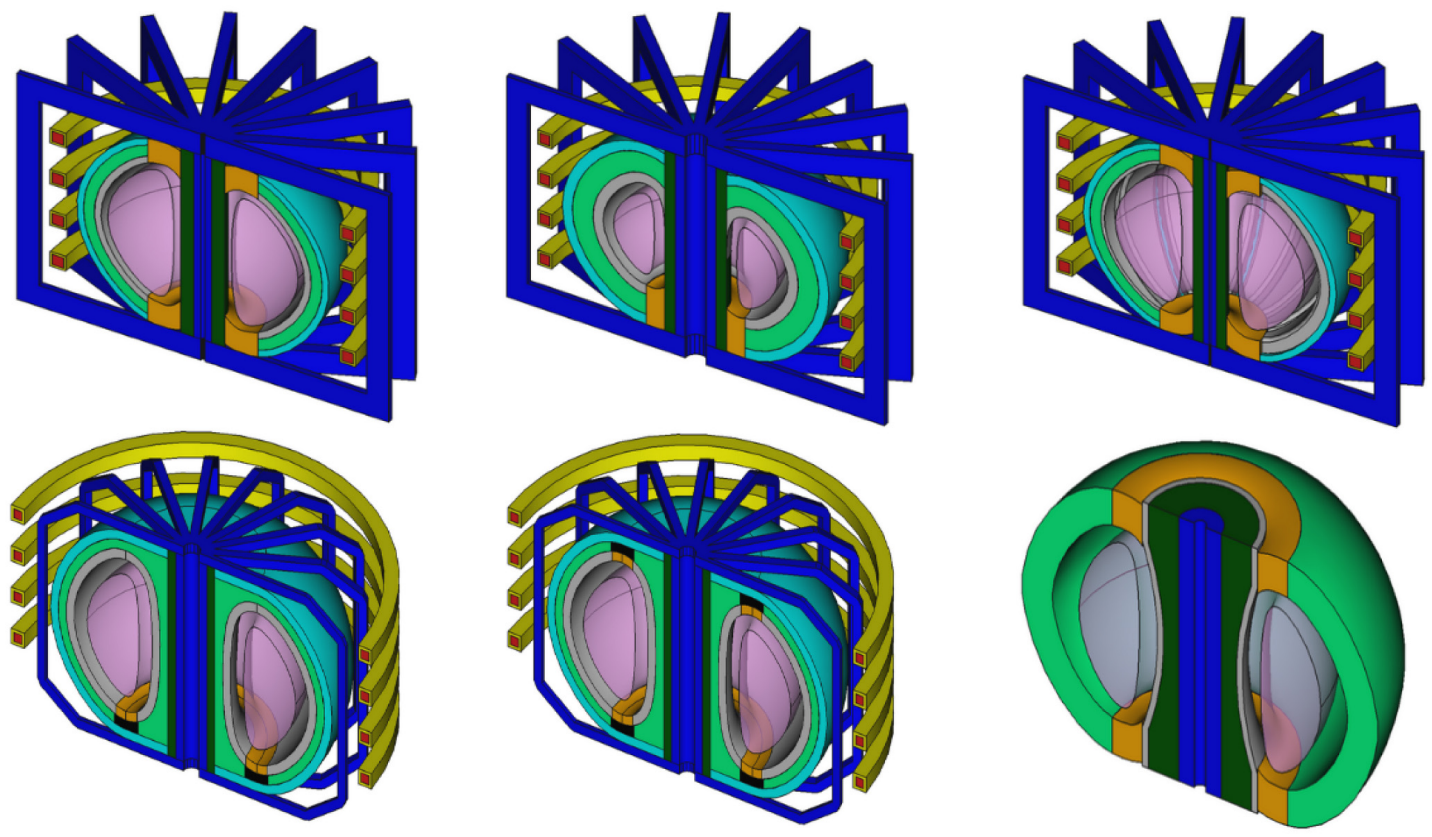
The current selection of reactors available. Note that because these reactors are all customisable with parameters they can appear differently to their default view pictured in the diagram. From left to right and up to down the reactor class names are BallReactor(), SingleNullBallReactor(), SegmentedBallReactor(), SingleNullSubmersionTokamak(), SubmersionTokamak() and CenterColumnStudyReactor().

There are currently over 34 Parametric Components available (see Figure 3) and many additional shapes are planned. When these components are combined then the variety of 3D volumes available is sufficient to start constructing simple fusion reactors as shown in Figure 4.

### Parametric Reactors

Parametric Reactors allow users to create a 3D reactor model by combining Parametric Components and Shapes with linkage that describes how they fit together. The models are not exact reproductions of any particular design but reflective of different reactor configurations that are available. There are currently six Parametric Reactors available in the Paramak (see Figure 4).

Two examples models created entirely from parameters are presented (see Figure 7 and Figure 8). In the case of the SegmentedBallReactor() the model has no inboard breeder zone and has divertors in the upper and lower positions. There are also single-null varieties of the BallReactor(). The main user inputs required are the radial thicknesses of components. The reactors require less user inputs than the individual components that make up the reactor would require. This is due to the radial build process that helps component inputs be derived from other components. The reactor design has the order of components encoded and therefore from this user information it is possible to know where each component starts and ends in the radial direction.

**Figure 5.**
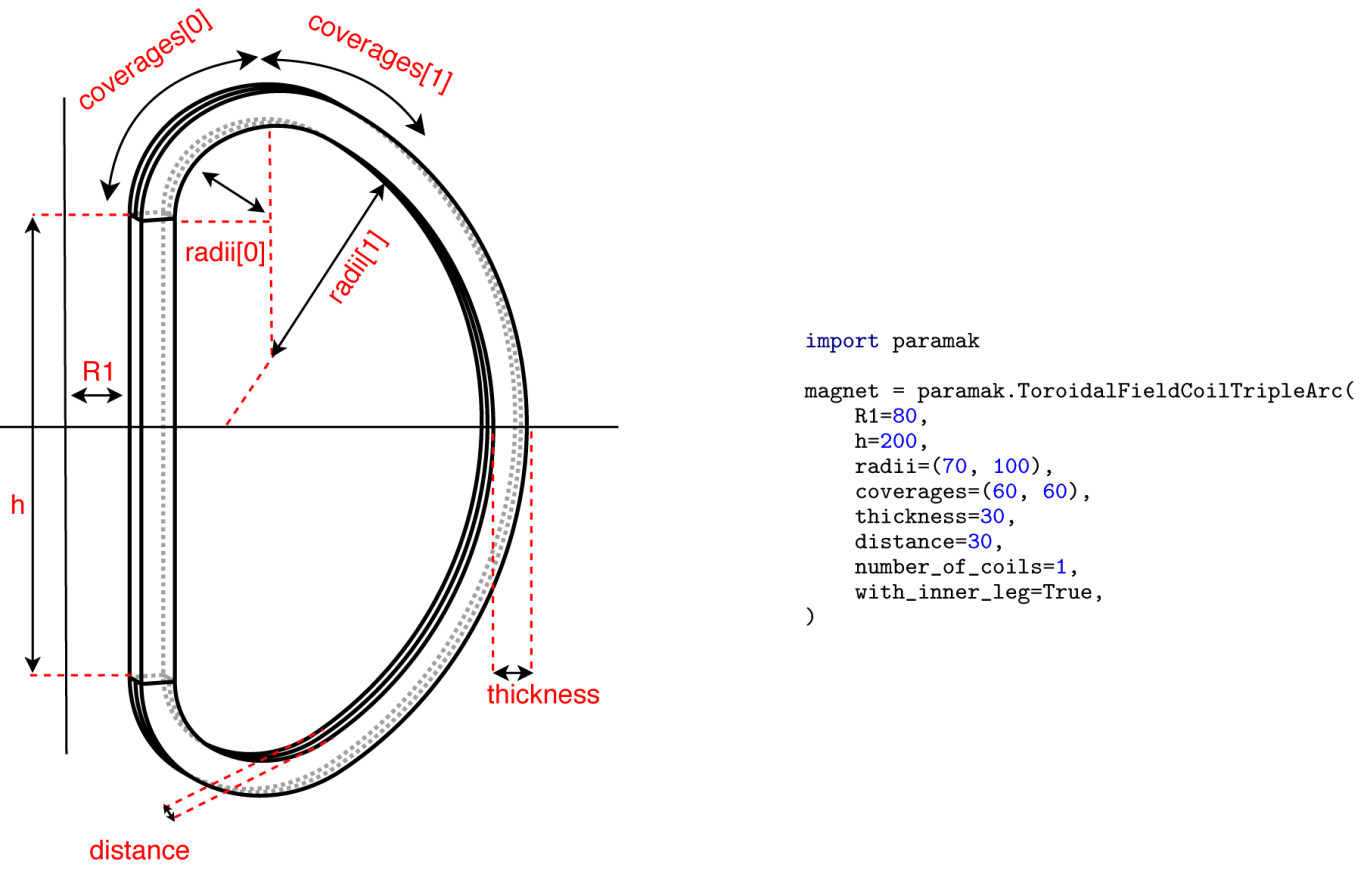
Example Parametric Component ToroidalFieldCoilTripleArc() with parameters labelled.

**Figure 6.**
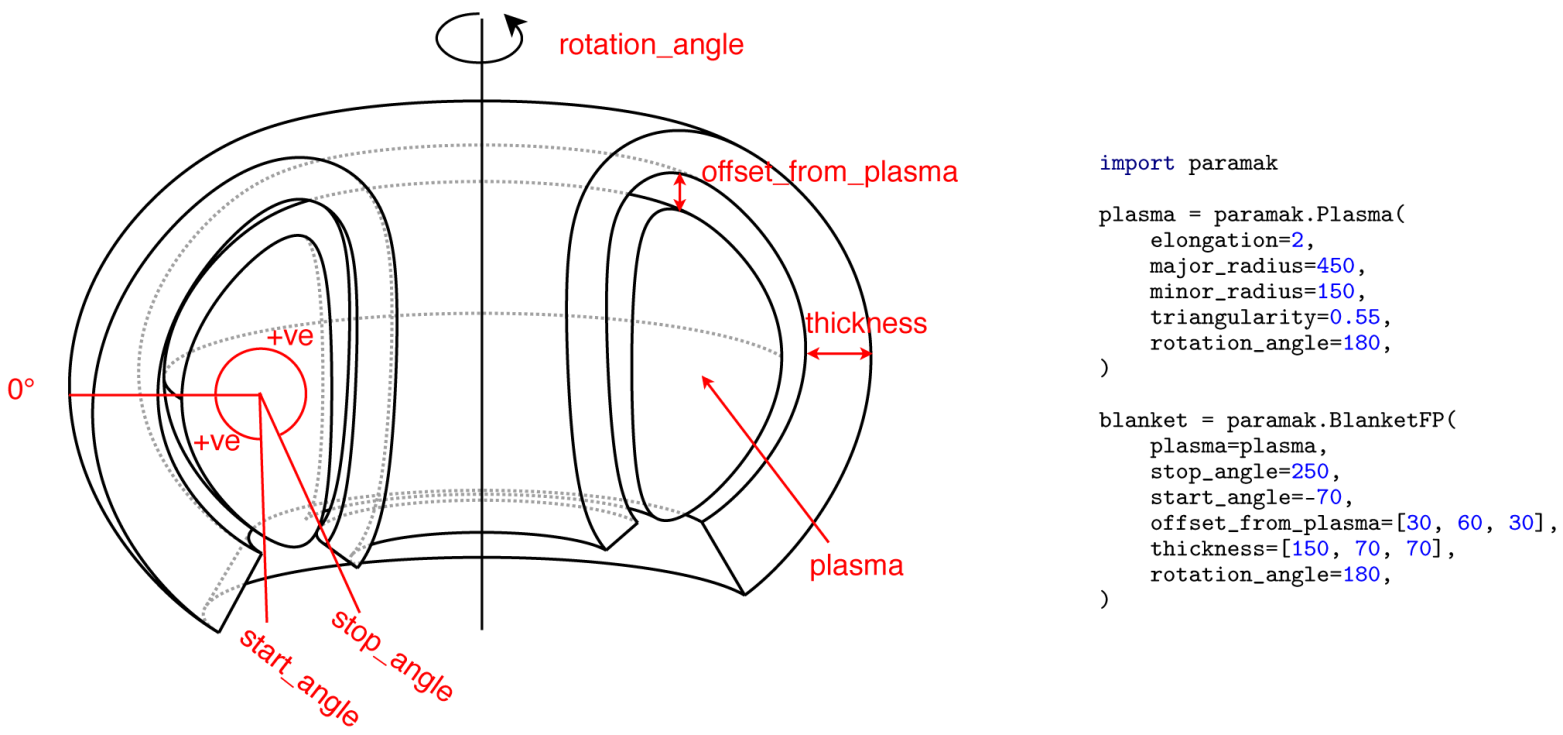
Example Parametric Component BlanketFP() build using a parametric Plasma as one of the inputs. Additionally the blanket has a variable thickness and variable offset from the plasma.

The vertical build for the SegmentedBallReactor() is largely based on the radial build which greatly minimises the number of user inputs required for a 3D model. The user inputs for the plasma elongation and triangularity, combined with the radial build parameters for the plasma, allow the coordinates of the top of the plasma to be calculated. The vertical offset from the firstwall to the plasma defaults to the same value as the outboard plasma gap radial thickness but can be specified independently using the plasma gap vertical thickness parameter. The blanket thickness is constant all around the reactor both in radial and vertical directions. The Parametric Component for the blanket accepts a variable thickness as a function of angle (see Figure 6) however this particular reactor design has been programmed to have constant thickness blankets throughout. This means the users will not be asked for a vertical blanket thickness but have less control over the reactor. The blanket is also segmented by another Parametric Component ( BlanketStarCutter()) to create banana segments. CadQuery’s inbuilt filter methods are then used to select the front edges of the firstwall and breeder zone so that they can be filleted. A Boolean cut between the firstwall block and the breeder zone results in a wrap around design. Positioning of poloidal field coils is a user controllable argument, however if (R,Z) coordinates are not specified then they are equispaced vertically behind the blanket. Four types of toroidal field coils exist as Parametric Components: rectangle, coat hanger, Princeton-D and triple arc. However, simple rectangular toroidal field coils are used for the current BallReactor() design. The SegmentedBallReactor() inherits from the BallReactor() so it also uses rectangular magnets by default. However other magnets shapes are also avaialble as parametric components (see Figure 5). When inheriting from a base design it is possible to overwrite any of the components. Due to this system the number of variations on the base design can rapidly increase. The BallReactor() design has inbuilt assumptions regarding the connections and shapes of components, this has disadvantages in terms of the flexibility but also the advantage of having reduced inputs for the user to specify.

**Figure 7.**
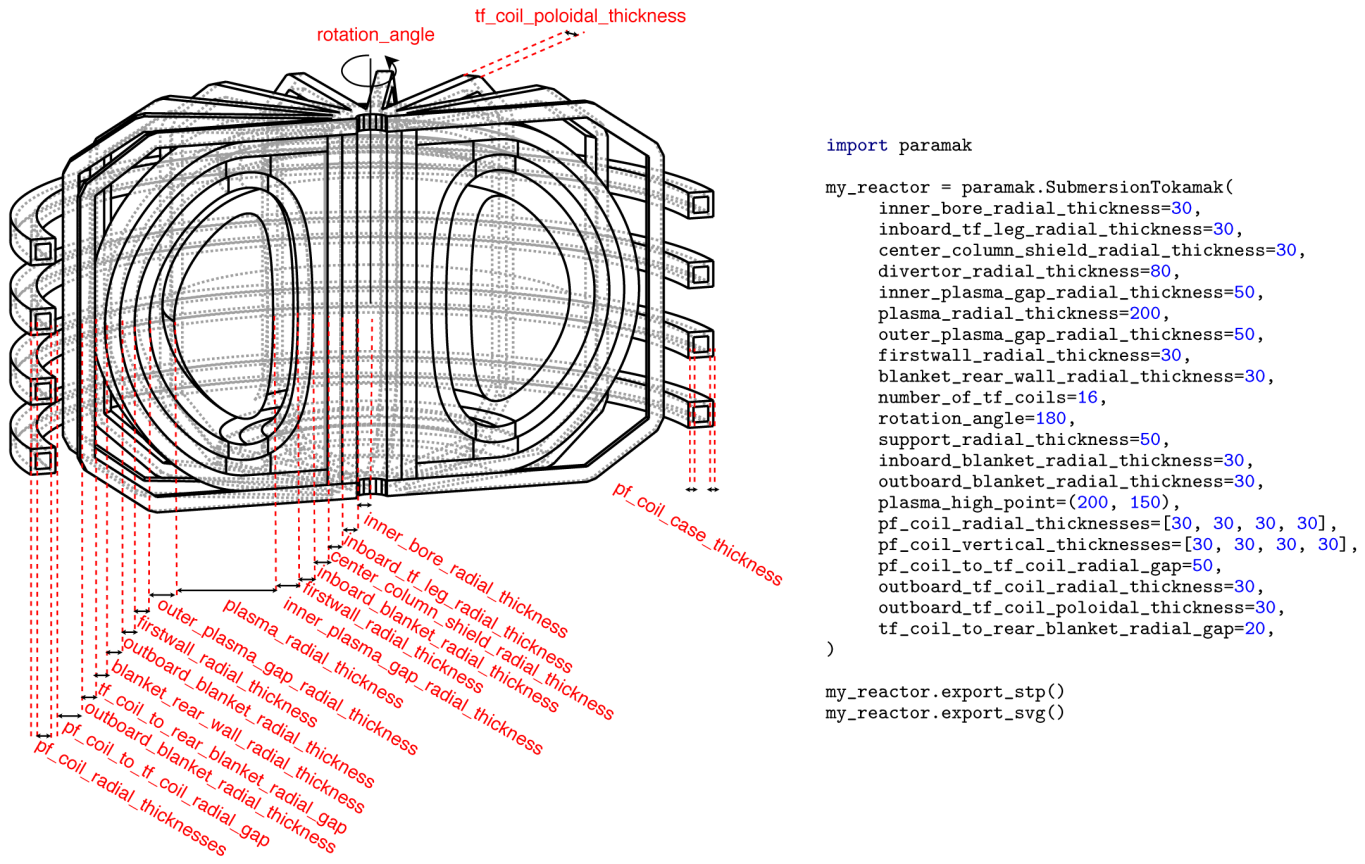
Example Python script showing the input parameters used for the creation of a SubmersionTokamak() model. The example also exports the SVG image used in this Figure and CAD files (STP) used when making Figure 4.

**Figure 8.**
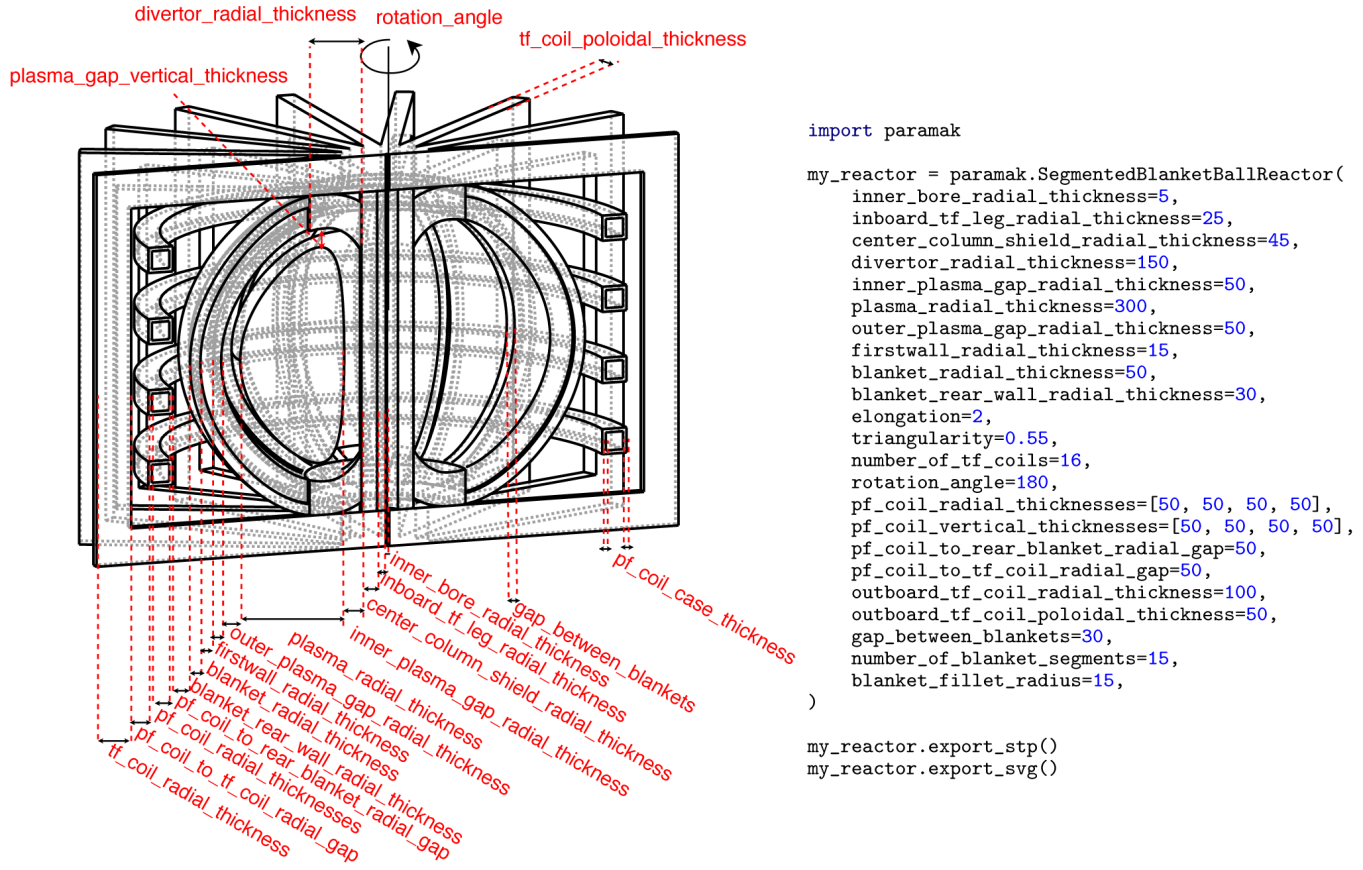
Example Python script showing the input parameters used for the creation of a SegmentedBallReactor() model. The example also exports the SVG image used in this Figure and CAD files (STP) used when making Figure 4.

The SubmersionTokamak() requires a few more inputs from the users and offers more flexibility when creating the models. Additional inputs are required for the radial thickness of the supports and the radial thickness of the inner blanket. The computational time to generate the 3D volumes and export CAD files in STP format once the input dimensions have been specified varies from around 20 seconds for a simple BallReactor() to around 40 seconds for a SegmentedBallReactor() on a desktop computer (i5 Intel processor). In this case the time difference is due to segmenting the blanket and filleting the edges of the blanket. Currently the entire construction process is a serial operation so there is scope to speed up the construction by parallelising parts of the construction process.

The CenterColumnStudy() reactor is designed for a specific use case. When study the impact of geometric parameters on the center column it is possible to simplify the design to only include components that significantly impact the simulation result. For example, the outboard TF and PF coils have little impact on the simulation results in this case. This reduces the time needed for model creation and reduces model initialisation in analysis use cases.

While the existing Parametric Reactors are not a full representation of magnetic fusion reactors, the framework established can be used to create more detailed components with more complex relationships between components.

All the various reactor classes allow operations such as exporting the volume(s) to CAD files (STP and STL format) and 2D images (SVG) of the geometry as used in the documentation ^12^. Other properties of the geometry can easily be obtained such as the volume of each shape or component in the reactor. This can be useful for cost estimates in systems codes or mass calculations in remote maintenance strategies. The utility of CAD models goes beyond visualisation and basic properties in assessing a design’s suitability and can be used as part of an automated parameter study. The Paramak knows the extent of the x, y, z dimensions for the geometry and therefore can automatically create thin shell bounding boxes (referred to as Graveyard volumes) for use in CAD based neutronics with DAGMC ^16^. While this paper aims to focus on the geometry creation within the Paramak there are future papers planned where utilisation within neutronics and engineering workflows will be demonstrated.

## Conclusion

The Paramak code has been introduced and the motivation for facilitating a data science approach to geometry construction has been discussed. Several benefits of the open-source approach have been realised during the project. The number of Parametric Components has grown to the level where simplified reactor models can be constructed. Reactor models can be encoded to encapsulate design decisions which allow the required user inputs to be limited. This is demonstrated by the three example models presented in the paper and reinforced by additional Parametric Reactor models contained in the documentation ^12^. There are currently six different Parametric Reactors for users to create. Due to the structure of the code, it is straightforward to inherit existing reactors and modify specific parts of their design to extend the reactor family to accommodate additional features or parameters of Parametric Reactors.

The current parametric models provided in the Paramak are relatively simple but it is also possible to make more complex models when provided with a design.

The Paramak has been used within UKAEA to create models of several spherical tokamak configurations and has also been used to reproduce a SPARC like design based on the diagrams in 17. The outputs of the Paramak are CAD models which are useful in fusion analysis disciplines such as Finite Element Analysis, neutronics, visualisation and even cost models which often require CAD files as an input.

Due to the use of modern software practices (continuous integration and containerisation), the software is able to test the CAD generated in neutronics analysis and demonstrate the compatibility of the geometry in use. The software employs modern software practices such as automatic documentation generation (ReadTheDocs) ^12^, package distribution services (PyPi) ^10^ and can be containerised ^11^. Consequently, the learning time, installation time and time to first results are minimal.

The use of these models in automated workflows has yet to be demonstrated in a publication but this would be the next logical stage in the process and the authors plan to publish a range of use cases for the parametric geometry in the future. Future work will, amongst other improvements, incorporate detailed parametric blanket models which have previously been created ^1^.

## Software availability

Paramak source code available from: https://github.com/ukaea/paramak

Archived source code as at time of publication: http://doi.org/10.5281/zenodo.4384269 ^18^

License: MIT
