# Supplementary material for: The Paramak: automated parametric geometry construction for fusion reactor designs
Source: F1000Res. 2021 Jan 18;10:27. [Version 1] doi: 10.12688/f1000research.28224.1 (PMC7983317; doi:10.12688/f1000research.28224.1)
Supplement: Peer review history

# Peer review: Reviewer response for version 1

**DOI:** 10.5256/f1000research.31218.r80436

**Published:** 2021-03-19

**Authors:** Jingang Liang

**Affiliations:** 1 Institute of Nuclear and New Energy Technology, Tsinghua University, Beijing, China

The article describes an open-source python package Paramak which facilitates rapid production of 3D CAD models of fusion reactors and helps parametric studies on geometry configurations for reactor prototyping. This tool, built upon an open-source CadQuery library and integrated with fusion reactor domain knowledge, provides an efficient framework for automated geometry creation and analysis of fusion reactor designs. It will be of interest to a wide range of researchers in nuclear engineering fields. In addition, Paramak employs modern software practices and open-source principles in its development, making it friendly to both users and developers.

 The manuscript is well-written. The methodology, implementation, and demonstrations are clear and reasonable. The source code of Paramak is available on GitHub, together with complete guides, examples, and other documentation. There is also an explanatory video that shows how to use the tool. The reviewer tried to install Paramak and tested some functionality. It worked well and as expected.

 I have no suggestions for improvement of the manuscript.

Are the conclusions about the tool and its performance adequately supported by the findings presented in the article?

Yes

Is the rationale for developing the new software tool clearly explained?

Yes

Is the description of the software tool technically sound?

Yes

Are sufficient details of the code, methods and analysis (if applicable) provided to allow replication of the software development and its use by others?

Yes

Is sufficient information provided to allow interpretation of the expected output datasets and any results generated using the tool?

Yes

Reviewer Expertise:

nuclear reactor physics; Monte Carlo particle transport; reactor benchmarks; reactor core design

I confirm that I have read this submission and believe that I have an appropriate level of expertise to confirm that it is of an acceptable scientific standard.

# Peer review: Reviewer response for version 1

**DOI:** 10.5256/f1000research.31218.r77632

**Published:** 2021-02-16

**Authors:** Yuefeng Qiu

**Affiliations:** 1 Institute for Neutron Physics and Reactor Technology, Karlsruhe Institute of Technology, Karlsruhe, Germany

Section "Integration in workflows": this section discusses two topics, one is on the preservation of the parametrization in the STEP format, another one is on software licensing. The author is suggested to discuss them in a separate paragraph to provide a better understanding. The licensing issues are not particularly concerned by the reader, and the discussion on licensing for a open-source software is confusing. 

 The ordering of the figures needs to be improved so that the reader doesn't need to jump over pages. For example, Figures 5/6 are mentioned before figures 3/4. 

 Section "parametric reactors": "The Parametric Component for the blanket accepts a variable thickness ... however this particular reactor design has been programmed to have constant thickness blankets throughout." The reviewer is not clear about this statement, whether the blanket thickness can vary or not. Because the blanket is essential to the fusion reactor design, providing users the necessary freedom of modifying the blanket is a key capability for this program. Please provide some examples of how the blanket thickness can vary in Figure 6, using thickness = [150,70,70] as an example. 

 Figure 4. the reviewer has seen the D shape of TF coil in Figure 1 but it hasn't been used for any of the six reactors in Figure 4. Please explain the reason. 

 Figure 7. Since the users are responsible for providing all the offsets and constraints of those components, the reviewer is interested in how the program can handle design failures, such as geometry collisions. This could possibly occur in the vertical direction because the geometry relation in the vertical direction seems not provided.

Are the conclusions about the tool and its performance adequately supported by the findings presented in the article?

Yes

Is the rationale for developing the new software tool clearly explained?

Yes

Is the description of the software tool technically sound?

Yes

Are sufficient details of the code, methods and analysis (if applicable) provided to allow replication of the software development and its use by others?

Partly

Is sufficient information provided to allow interpretation of the expected output datasets and any results generated using the tool?

Yes

Reviewer Expertise:

nuclear physics, fusion neutronics

I confirm that I have read this submission and believe that I have an appropriate level of expertise to confirm that it is of an acceptable scientific standard.
